# On the Causes and Prevention of Apoplexy and Paralysis

**Published:** 1845-12

**Authors:** Marshall Hall


*On the Causes and Prevention of Apoplexy and Paralysis*—By Marshall Hall, M.D., F.R.S.—There is no more delicate or more momentous question in the practice of medicine, than that of the prevention of the attacks of apoplexy, hemiplegia, or other paralytic affections. These arise from such different and even opposite causes, that the very course of medicine and regimen which is most conducive to safety in one case, has the opposite tendency in another; wherefore, the diagnosis on which our treatment depends requires the utmost care and attention.

There is sometimes a state of plethora, sometimes of anæmia, and in other cases, there is neither the one nor the other, but that morbid condition of the system, called cachexia, often denoted by boils, carbuncles, &c., with deranged secretions—as the predisposing cause of attacks of paralysis.

Allied to this last condition, is that which obtains in dyspepsia and gout. The imperfect performance of the function of the liver, and especially of the kidneys, is in another class of cases, the cause of these formidable diseases. In other cases there is disease of the heart, or of the minute arteries and veins, or of the capillary vessels of the brain and its membranes.

The attention of physicians has, until recently, been too much directed to fulness as the general cause of the apoplectic or paralytic attack; and as to the public, they have to this moment only one idea—that a fit denotes fulness; and the practitioner, who, on being summoned to such a case, shall brave this opinion, and, depending on his professional knowledge, discard that invariable refuge of the timid and ignorant, the lancet, will make himself responsible for the issue.

The author, after stating that the real principle upon which an apoplectic seizure is to be prevented, is to induce a state of equilibrium in regard to plethora or inanition, and of health in regard to the general tone, habit, and secretions, proceeds to remark upon each of the conditions to which he before adverted; and first, of

1. *Plethora.*—When plethora is the cause of the threatening of apoplexy the remedy and safety of the patient consists in depletion. How are we to be certain of the fact (of plethora)? There may be the appearance of the sanguineous temperament, an athletic form, &c.; and with all this, there may be headache, vertigo, and other symptoms of head affection. But is it certain that the symptoms in such a case depend upon fulness? If there be, in addition to the symptoms enumerated, a disposition to *doze*, it is nearly so. But in the absence of such symptom, and even with such symptom, may not the real case be indigestion? Certainly; then what is to be done?

There is a [diagnostic] symptom of great value, when it can be clearly ascertained to exist. It is the occurrence of vertigo, first, in the act of stooping, and secondly, in an unusually erect posture, especially when suddenly assumed. But if this be absent, what is then to be done?

There is a resource in such a case, which, in spite of the criticism of a respectable author, I will again venture to assert is of immense value. There is no case in which a patient, if bled from a good orifice, in the erect posture, bears to lose so much blood before syncope takes place, as in that of real congestion of the cerebral vessels; there is no case in which full abstraction of blood is so necessary. On the other hand, in the case of vertigo, and other cerebral symptoms arising from dyspepsia, the patient neither bears the loss of much blood, nor requires it.

In a doubtful case, I propose to adopt this mode of bloodletting, first, as a guard against the undue loss of blood, and secondly, as a means of *diagnosis*, and a prompter of ulterior proceedings. I have adopted this measure so often, and with such satisfactory results, that I cannot recommend it too strongly to my medical brethren. In cases, on the other hand, in which it has not been adopted, I have seen one class of patients become a prey to apoplectic or paralytic seizures, for want of bloodletting, and another affected with headache and vertigo, drained of blood (uselessly) by repeated cupping and leeches.

2. *Anæmia.*—I now come to speak of cases in which anæmia has already been induced. This is not a state of immunity even against an attack of paralysis or apoplexy. I constantly see patients who are in jeopardy, not from fulness, but from inanition, and who have been kept in a state of anæmia by bloodletting, general or local, when an opposite treatment is required to restore the equilibrium of the system.

A state of pallor; a disposition to vertigo, faintishness, palpitation; a state of nervous timidity; the recurrence of the symptoms when the stomach is empty, when the bowels are freely moved, or upon looking upwards, or upon resuming the upright position after stooping or rising from the bed—such are the diagnostic signs of a state of inanition from that of plethora.

The history of the case is in every respect a great aid in the diagnosis. If depletion has been used, it has been attended with this result—temporary alleviation, with subsequent aggravation of all the symptoms; an opposite mode of treatment, very cautiously adopted, and prudently pursued, involving quinine and iron, confers a more permanent, but less immediate benefit.

The state of anæmia itself is not without danger even of apoplexy or paralysis; the actual effusion of blood having occurred in some instances. Such a case is related by Denman; it occurred in the midst of exhaustion from hemorrhage from uterine polypus. We might, therefore, incautiously bleed our patient into hemiplegia.

3. *Dyspepsia—Cachexia.*—I have so often observed symptoms threatening apoplexy in conjunction with symptoms of dyspepsia and cachexia, that I have no doubt of the importance of a strict attention to the subject. One form is the following: vertigo occurs with faintishness, nausea, and cold clammy perspiration; sometimes there is actual sickness and much flatus. In these cases the liver is torpid, and the urine is apt to deposit lithic acid salts. In no case is the loss of blood repaired with such difficulty. The application of a few leeches sometimes leaves a state of debility and pallor for weeks. The treatment consists in the correction of the secretion, and in giving tone to the system. The compound decoction of aloes, infusion of rhubarb, of gentian, and sarsaparilla, vinum ferri, and the bicarbonate of potash, are the principal remedies; but with these, a mild nutritious diet, a system of gentle exercises, early hours, and a strict attention to the condition of the feet and general surface, should be conjoined.

4. *Gout.*—There is frequently a connexion to be traced between gout and its frequent attendant the lithic acid diathesis, and the apoplectic and hemiplegic seizure. The steady perseverance in such remedies as the decoct. aloes compos., bicarbonate of potash, &c., have in many cases averted the evil. The vinum colchici should also be given, in very minute doses, as five drops three times a day, to overcome the specific gouty diathesis.

The lithic acid diathesis is not the only urinary disorder which leads to apoplexy and hemiplegia; the attack, as it is well known, occurs in diabetes, and in the case of albuminous urine.

Such are the predisposing causes of apoplexy and paralysis; the prevention must consist in removing them; and, according to circumstances, depletion, iron, sarsaparilla, colchicum, &c., must be prescribed, and steadily persevered in.—*Rank. Abs.*

